# MicroRNAs as the critical regulators of autophagy-mediated cisplatin response in tumor cells

**DOI:** 10.1186/s12935-023-02925-7

**Published:** 2023-04-25

**Authors:** Faezeh Tolue Ghasaban, Amirhosein Maharati, Iman Akhlaghipour, Meysam Moghbeli

**Affiliations:** 1 Department of Medical Genetics and Molecular Medicine, School of Medicine, Mashhad University of Medical Sciences, Mashhad, Iran; 2 Student Research Committee, Faculty of Medicine, Mashhad University of Medical Sciences, Mashhad, Iran

**Keywords:** Autophagy, Cisplatin, Chemotherapy, Drug resistance, Cancer, microRNA

## Abstract

Chemotherapy is one of the most common therapeutic methods in advanced and metastatic tumors. Cisplatin (CDDP) is considered as one of the main first-line chemotherapy drugs in solid tumors. However, there is a high rate of CDDP resistance in cancer patients. Multi-drug resistance (MDR) as one of the main therapeutic challenges in cancer patients is associated with various cellular processes such as drug efflux, DNA repair, and autophagy. Autophagy is a cellular mechanism that protects the tumor cells toward the chemotherapeutic drugs. Therefore, autophagy regulatory factors can increase or decrease the chemotherapy response in tumor cells. MicroRNAs (miRNAs) have a pivotal role in regulation of autophagy in normal and tumor cells. Therefore, in the present review, we discussed the role of miRNAs in CDDP response through the regulation of autophagy. It has been reported that miRNAs mainly increased the CDDP sensitivity in tumor cells by inhibition of autophagy. PI3K/AKT signaling pathway and autophagy-related genes (ATGs) were the main targets of miRNAs in the regulation of autophagy-mediated CDDP response in tumor cells. This review can be an effective step to introduce the miRNAs as efficient therapeutic options to increase autophagy-mediated CDDP sensitivity in tumor cells.

## Background

Chemotherapy, radiotherapy, and surgery are the most prominent clinical techniques in cancer therapy [1]. Since, chemotherapeutic medications can reach every organ in the body through the circulation, it is recognized as the most beneficial treatment option for the majority of patients with late-stage and metastatic cancer [2]. Despite pharmacological advances in tumor therapy, the emergence of multidrug resistance (MDR) limits the efficiency of chemotherapy in tumor cells [3, 4]. MDR is associated with genetic and growth factors, increased DNA repair capability, drug efflux, and xenobiotic metabolism [5]. Platinum-based drugs inhibit DNA replication and transcription, resulting in cell cycle arrest and apoptosis, through covalently interacting with purine bases to construct interstrand and intrastrand DNA adducts [6]. Cisplatin (CDDP) is an inorganic chemotherapeutic agent frequently used to treat diverse malignancies that has the potential to significantly raise the overall survival rates of cancer patients [7, 8]. It is a highly effective anticancer drug that is frequently utilized in the first-line treatment of solid tumors [9]. However, Cisplatin treatment is frequently accompanied with various side effects, such as nausea, vomiting, alopecia, liver disorders, and bone marrow inhibition [10]. Although, the majority of cancer patients respond to platinum, cisplatin-resistance and tumor relapse can be observed among cancer patients [9]. It has been discovered that half of the patients treated with cisplatin acquire multidrug or intrinsic resistance [9, 11]. Therefore, clarifying the molecular mechanisms to overcome cisplatin resistance can greatly enhance prognosis. Drug efflux, drug uptake, DNA repair, autophagy, and apoptosis are the key regulators of cisplatin resistance [12]. Autophagy disruption increases CDDP-mediated apoptosis in various cancer cells [13–15].

Autophagy is a conserved catabolic mechanism of organelle recycling that has a cytoprotective role against adverse conditions including nutrient deprivation, reactive oxygen species, and cell stress [16, 17]. Autophagy is the process by which autophagosomes wrap damaged cell components in bilayer lipid vesicles and then destruct them via lysosomal fusion [18]. It is involved in regulation of a variety of biological processes, including cell survival, differentiation, cell death, and tumorigenesis [19, 20]. Autophagy deregulation has been linked to a variety of diseases, such as diabetes, cardiomyopathy, and cancer [21–23]. It is a cell survival strategy that confers tumor cell survival and poor prognosis through induction of dormancy during therapeutic phases [24]. Therefore, its activation allows drug-resistant tumors to maintain their viability [25]. Autophagy suppression paired with chemotherapy resulted in enhanced tumor cell death, suggesting its pro-survival function in the development of chemotherapy resistance [26].

MicroRNAs (miRNAs) are a category of small non-coding RNAs that function as the negative post-transcriptional regulators by mRNA degradation or translational inhibition [27, 28]. They are involved in regulation of cell proliferation, migration, and cell death [29, 30]. MiRNAs have also been implicated in the tumor progression and chemo resistance [31, 32]. Since, circulating miRNAs are highly stable in urine and blood, they can be used as effective and non-invasive tumor markers [33–35]. MiRNAs are involved in autophagy by regulation of retrieval stages, induction, vesicle elongation, and vesicle nucleation [36]. They also play a role in the cisplatin response of tumor cells by regulation of autophagy [34]. Regarding the side effects of cisplatin on healthy tissues, it is required to clarify the molecular mechanisms of cisplatin resistance to provide novel efficient therapeutic modalities to reduce the side effects of chemotherapy in cancer patients. Accordingly, microRNAs as the non-invasive and more stable factors compared with mRNAs, can be introduced as valuable prognostic markers of cisplatin response in cancer patients through autophagy regulation. Taken together, considering the importance of the autophagy in response to cisplatin treatment, in the present review we discussed the role of miRNAs in regulation of autophagy-mediated cisplatin response in tumor cells (Table 1).

**Table 1:** Role of miRNAs in autophagy-mediated cisplatin response

miRNA	Type	Target	Samples	Autophagy	CDDP response	Clinical application	Study
Signaling pathways							
miR-181	Non-small cell lung cancer	PI3K/Akt/mTOR pathway	6T 6 N* A549/DDP cell line	Induction	Increased CDDP sensitivity	Diagnosis	Liu [42]
miR-22	Osteosarcoma	PI3K/Akt/mTOR pathway	MG-63, MG-63/CDDP, U2OS, Saos2 and OS9901 cell lines Xenograft model	Inhibition	Increased CDDP sensitivity	Diagnosis	Meng [43]
miR-5047	Cervical canecr	VEGFA	25T 25 N HeLa and SiHa cell lines	Inhibition	Increased CDDP sensitivity	Diagnosis	Guo [50]
miR-99a-5p	Gastric cancer	MTMR3	25R 75 S* BGC823, SGC7901, and SGC7901CDDP cell lines Xenograft model	Induction	Increased CDDP resistance	Diagnosis	Sun [51]
miR-339-5p	Laryngeal carcinoma	TAK1	Hep-2 cell line	Inhibition	Increased CDDP sensitivity	Diagnosis	Li [52]
miR-222	Bladder cancer	PPP2R2A	T24 and 5637 cell lines	Inhibition	Increased CDDP resistance	Diagnosis	Zeng [60]
miR-26a	Glioblastoma	GSK3β	U87MG and U251MG cell lines	Induction	Increased CDDP resistance	Diagnosis	Ma [65]
miR-205	Nasopharyngeal carcinoma	HER3	CNE1, CNE2, SUNE1, and HK1 cell lines	Induction	Increased CDDP sensitivity	Diagnosis	Hao [67]
miR-144	Anaplastic thyroid carcinoma	TGF-α	5T 5 N ARO and TPC1 cell lines Xenograft model	Inhibition	Increased CDDP sensitivity	Diagnosis	Liu [69]
miR-144-3p	Anaplastic thyroid carcinoma	TGF-α	5T 5 N TPC1 and BHT101 cell lines Xenograft model	Inhibition	Increased CDDP sensitivity	Diagnosis	Liu [70]
miR-145-5p	Laryngeal squamous cell carcinoma	PRKCI	FD-LSC-1 and Tu 177 cell lines Xenograft model	Induction	Increased CDDP sensitivity	Diagnosis	Gao [73]
miR-425-3p	Non-small cell lung cancer	AKT1	19R 19 S 15 after first trial 15 after last trial A549 and A549/DDP cell lines	Induction	Increased CDDP resistance	Diagnosis and prognosis	Ma [76]
miR-30a	Ovarian/ breast/ liver cancer	Beclin-1	HeLa, MCF-7, HepG2, and HepS cell lines Xenograft model	Inhibition	Increased CDDP sensitivity	Diagnosis	Zou [81]
miR-30a-5p	Small Cell Lung Cancer	Beclin-1	22R 32 S H446 and Letp cell lines	Inhibition	Increased CDDP sensitivity	Diagnosis	Yang [82]
miR-216b	Non-small cell lung cancer	Beclin-1	40T 40 N A549 cell line Xenograft model	Inhibition	Increased CDDP sensitivity	Diagnosis	Chen [83]
miR-148a-3p	Gastric cancer	AKAP1/RAB12	105T 105 N BGC823CDDP, SGC7901CDDP, BGC823, and SGC7901 cell lines Xenograft model	Inhibition	Increased CDDP sensitivity	Diagnosis and prognosis	Li [90]
miR-136-5p	Laryngeal squamous cell carcinoma	ROCK1	FD-LSC-1 and FaDu cell lines	Induction	Increased CDDP sensitivity	Diagnosis	Yang [95]
miR-142-3p	Gastric cancer	ROCK2	100T 100 N 48 GC blood sample 48 healthy blood sample AGS, SGC-7901, MKN-45, and BGC-823 cell lines xenograft model	Inhibition	Increased CDDP resistance	Diagnosis and prognosis	Peng [97]
Apoptosis and drug efflux							
miR-15a-3p	Non-small cell lung cancer	BCL2	Calu1 cell line	Induction	Increased CDDP sensitivity	Diagnosis	Bozok [105]
miR-143	Cervical cancer	BCL2	HeLa and CaSki cell lines	Induction	Increased CDDP sensitivity	Diagnosis	Esfandyari [106]
miR-7-5p	Cervical cancer	PARP-1/Bcl-2	15T 15 N HeLa and SiHa cell lines	Induction	Increased CDDP resistance	Diagnosis	Yang [107]
miR-30a	Oral squamous carcinoma	Beclin1	13T 14 N SCC084 cell line	Inhibition	Increased CDDP sensitivity	Diagnosis	Kulkarni [108]
miR-30	Gastric cancer	LC3II/ LC3-I	SGC-7901 cell line	Inhibition	Increased CDDP sensitivity	Diagnosis	Du [111]
miR-22	Osteosarcoma	caspase-3/ Bcl-2/ ATG5/ beclin1/ LC3B	MG-63, MG-63/CDDP, U2OS, Saos2 and OS9901 cell lines Xenograft model	Inhibition	Increased CDDP sensitivity	Diagnosis	Meng [116]
miR-22	Osteosarcoma	MTDH	MG-63 cell line	Inhibition	Increased CDDP sensitivity	Diagnosis	Wang [117]
Ubiquitin-like modifiers and autophagy receptors							
miR-199a-5p	Small cell lung cancer	p62	30T 30 N NCI-H446 and H69PR cell lines Xenograft model	Induction	Increased CDDP resistance	Diagnosis	Li [124]
miR-146a	Lung cancer	CHOP	69T 69 N A549 and H446 cell lines Xenograft model	Inhibition	Increased CDDP resistance	Diagnosis	Tan [131]
miR-133a	Ovarian cancer	YES1	24R 12 S SKOV3 and A2780 cell lines Xenograft model	Inhibition	Increased CDDP sensitivity	Diagnosis	Zhou [134]
Transcription factors and DNA binding proteins							
miR-29c-3p	Ovarian cancer	FOXP1/ATG14	SKOV3 and A2780 cell lines Xenograft model	Inhibition	Increased CDDP sensitivity	Diagnosis	Hu [136]
miR-125b	Thyroid cancer	ATG7/ Foxp3	30T 30 N WRO, FRO, and KAT18 cell lines Xenograft model	Induction	Increased CDDP sensitivity	Diagnosis	Wang [137]
miR-152	Ovarian cancer	ATG14	35T A2780/CP70, SKOV3/DDP, A2780 and SKOV3 cell lines Xenograft model	Inhibition	Increased CDDP sensitivity	Diagnosis	He [139]
miR-579-3p	Osteosarcoma	MSH6	SCSP-5030 and TCHu124 cell lines Xenograft model	Inhibition	Increased CDDP sensitivity	Diagnosis	Zhan [145]
miR-216b	Non-small cell lung cancer	Beclin-1	40T 40 N A549 and cisplatin resistance A549/DDP cell lines Xenograft model	Inhibition	Increased CDDP sensitivity	Diagnosis	Chen [151]
miR-181a-5p	Breast cancer	VDR	HS578T, HCC70, MDA-MB-231, MDA-MB-468 and BT549 cell lines	Induction	Increased CDDP sensitivity	Diagnosis	Lin [154]
Autophagy-related genes							
miR-1278	Nasopharyngeal carcinoma	ATG2B	90T 90 N CNE-1, CNE-2, C666-1, 5–8 F and HONE-1 cell lines Xenograft model	Inhibition	Increased CDDP sensitivity	Diagnosis and prognosis	Zhao [161]
miR-376a	laryngocarcinoma	ATG2A	30T 30 N SNU46 and M4E cell lines Xenograft model	Inhibition	Increased CDDP sensitivity	Diagnosis	Feng [163]
miR-1	Non-small cell lung cancer	ATG3	30R 30 S A549 and H1299 cell lines	Inhibition	Increased CDDP sensitivity	Diagnosis	Hua [164]
miR-651	Cervical canecr	ATG3	30T 30 N 30T blood sample 30 N blood sample C33A, HT-3, HeLa/S, and HeLa/DDP cell lines	Inhibition	Increased CDDP sensitivity	Diagnosis and prognosis	Zhu [165]
miR-16	Osteosarcoma	ATG4B	30T 30 N SAOS2 and U2OS cell lines	Inhibition	Increased CDDP sensitivity	Diagnosis	Liu [168]
miR-101-3p	Non-small cell lung cancer	ATG4D	A549, PC-9, NCI-H1299, and HCC827 cell lines Xenograft model	Inhibition	Increased CDDP sensitivity	Diagnosis	Cui [169]
miR-101	Hepatocellular carcinoma	RAB5A/ STMN1/ ATG4D	HepG2 cell lines	Inhibition	Increased CDDP sensitivity	Diagnosis	Xu [170]
miR-30e	Gastric cancer	ATG5	SGC7901 cell line Xenograft model	Inhibition	Increased CDDP sensitivity	Diagnosis	Zhang [173]
miR-181a	Gastric cancer	ATG5	SGC7901/CDDP cell line Xenograft model	Inhibition	Increased CDDP sensitivity	Diagnosis	Zhao [174]
miR-1301	Ovarian cancer	ATG5/ Beclin1/ EMT	SKOV3 cell line	Inhibition	Increased CDDP sensitivity	Diagnosis	Yu [175]
miR-30b	Gastric cancer	ATG5	AGS, HGC-27 and the 293T cell lines	Inhibition	Increased CDDP sensitivity	Diagnosis	Xi [176]
miR-17	Non-small cell lung cancer	ATG7	50T 50 N A549 and H1299 cell lines	Inhibition	Increased CDDP sensitivity	Diagnosis	Sun [180]
miR-138-5p	Non-small-cell lung cancer	TRIM65	30R 30 S A549 and A549/DDP cell lines Xenograft model	Inhibition	Increased CDDP sensitivity	Diagnosis	Pan [185]
miR-199a-5p	Hepatocellular carcinoma	ATG7	21T blood sample before and after treatment Huh7 cells, HepG2 cell lines	Inhibition	Increased CDDP sensitivity	Diagnosis	Xu [186]
miR-654-5p	Non-small cell lung cancer	ATG7	108T 108 N H1975, H820, H1299, H358, and A549 cell lines	Inhibition	Increased CDDP sensitivity	Diagnosis	Kong [187]
miR-17	Nonsmall cell lung cancer	ATG7	A549 and H1299 cell lines Xenograft model	Inhibition	Increased CDDP sensitivity	Diagnosis	Huang [188]
miR-7	Bladder cancer	ATG7	47T 47 N T24T and EJ cell lines Xenograft model	Inhibition	Increased CDDP sensitivity	Diagnosis	Wang [189]
miR-646	Oral squamous cell carcinoma	ATG13	SCC-15 and CAL-27 cell lines Xenograft model	Inhibition	Increased CDDP resistance	Diagnosis	Gao [190]
miR-186	Colorectal cancer	ATG14	50T 50 N SW620 and SW480 cell lines Xenograft model	Inhibition	Increased CDDP sensitivity	Diagnosis	Han [191]
miR-410	Osteosarcoma	ATG16L1	40T 40 N U2OS and MG-63 cell lines	Inhibition	Increased CDDP sensitivity	Diagnosis and prognosis	Chen [195]
Structural factors and enzymes							
miR-205	Prostate cancer	RAB27A/LAMP3	DU145 and PC-3 PCa cell lines Xenograft model	Inhibition	Increased CDDP sensitivity	Diagnosis	Pennati [199]
miR-140-5p	Non-small cell lung cancer	WEE1	30R 30 S A549 and H1299 cell lines Xenograft model	Inhibition	Increased CDDP sensitivity	Diagnosis	Fu [200]
miR-329-3p	Neuroblastoma	MYO10	26T 26 N HUVEC and NB cell lines	Inhibition	Increased CDDP sensitivity	Diagnosis	Wang [203]
miR-124/ miR-142	Non-small cell lung cancer	SIRT1	36T 36 N H1299 and H1299/CDDP cell lines Xenograft model	Inhibition	Increased CDDP sensitivity	Diagnosis	Song [208]

*Tumor (T) tissues and Normal (N) margins*Resistant (R) patients and Sensitive (S) patients to CDDP

### Role of miRNAs in autophagy-mediated CDDP response by regulation of signaling pathways

**Fig. 1:**
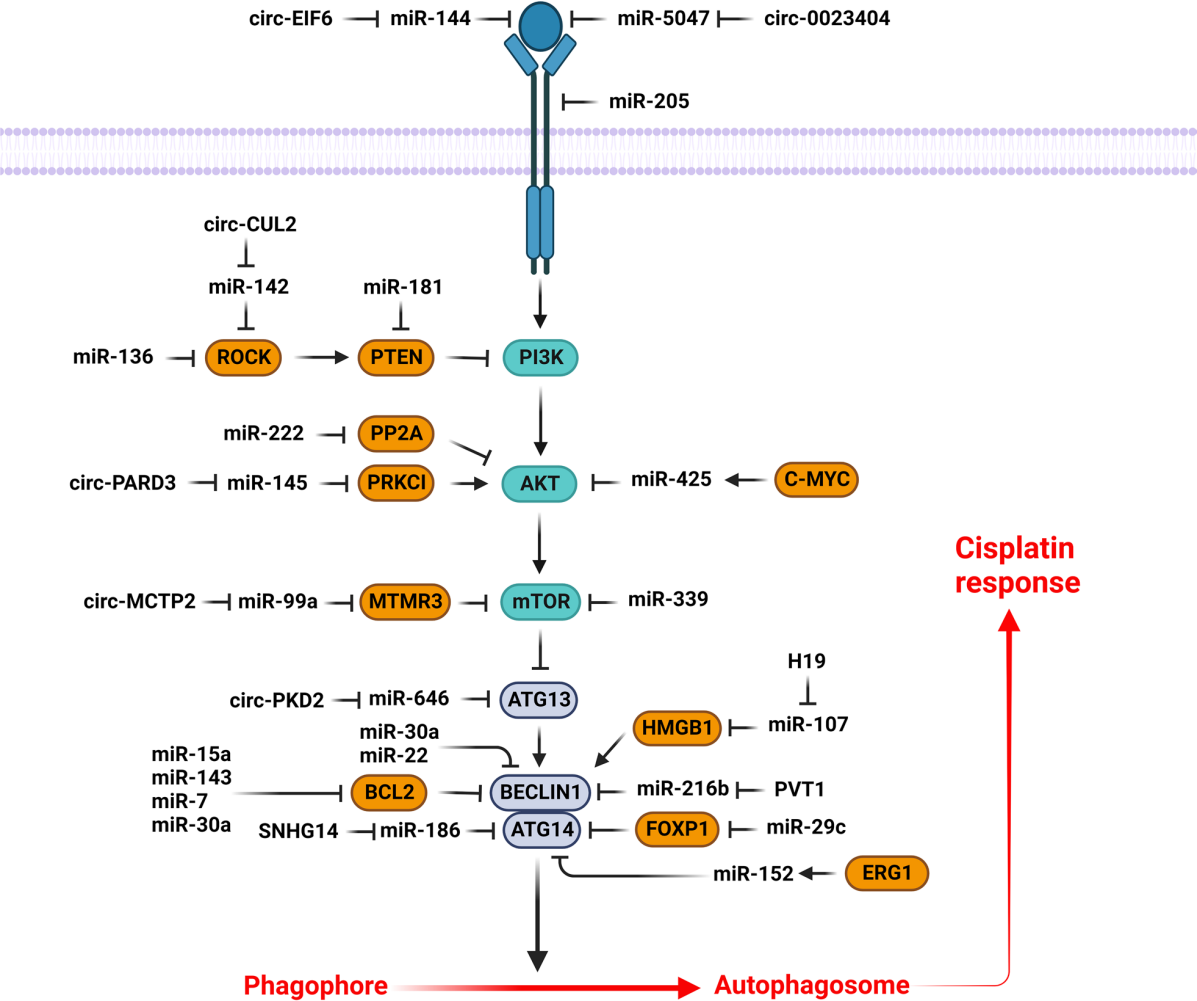
Role of miRNAs in autophagy-mediated cisplatin response via regulation of PI3K/AKT signaling pathway. (Created with BioRender.com)

The PI3K/AKT/mTOR signaling pathway promotes the tumor progression by increased cell proliferation while reduced autophagy. MicroRNAs have a pivotal role in autophagy-mediated cisplatin response by regulation of PI3K/AKT signaling pathway (Fig. 1). PTEN is a crucial stimulator of autophagy by PI3K/PKB inhibition [37, 38]. Despite the recent improvements in Non-small-cell lung cancer (NSCLC) treatment, there is still a poor prognosis in advanced stage patients with an overall survival rate of 15% that can be associated with chemoresistance and tumor relapse [39, 40]. Cisplatin is one of the primary post-surgical adjuvant therapeutics for NSCLC [41]. It was shown that miR-181 was significantly down regulated in cisplatin-resistant NSCLC patients compared to healthy controls. MiR-181 inhibition was associated with Autophagy related 5 (ATG5) and Microtubule-associated protein light chain 3 (LC3) down regulations in A549/DDP cells. MiR-181 reduced cell proliferation while promoted autophagy via the PTEN/PI3K/AKT/mTOR pathway in A549/DDP cells [42]. MiR-22 repressed cisplatin resistance in osteosarcoma via autophagy suppression through the PI3K/AKT/mTOR axis [43]. MTMR3 is an inositol lipid 3-phosphatase from the myotubularin family that hydrolyzes the Phosphatidylinositol 3-phosphate (PI3P) autophagic effector [44, 45]. The autophagosome formation has been shown to be repressed by MTMR3-mediated inhibition of the PI3P [46]. It is also a negative regulator of mTORC1 [47]. Circular RNAs (circRNAs) are covalently closed loop non-coding RNAs lacking either polyadenylation or 5′ to 3′ polarity [48]. They are involved in regulation of cell proliferation, migration, apoptosis, autophagy, and chemoresistance [49, 50]. It has been shown that there was circMCTP2 down regulation in CDDP-resistant gastric cancer (GC) cells. CircMCTP2 enhanced CDDP sensitivity via sponging miR-99a-5p and upregulating MTMR3 in GC cells [51]. MiR-339-5p has been reported to reduce CDDP resistance of laryngeal carcinoma by hindering the autophagy process through TAK1 targeting. MiR-339-5p also down regulated the mTOR and AMPK in CDDP-resistant cells [52].

The phosphatase 2 A (PP2A) is a pivotal regulator of cell cycle, metabolism, protein synthesis, and cell death [53]. PP2A downregulation has been associated with increased tumor progression via induction of various proliferative kinases in cancer cells [54, 55]. PPP2R2A is a member of regulatory B subunits of the PP2A family [56]. It was found to be downregulated by miR-222, which activated the AKT/mTOR axis [57–59]. MiR-222 enhanced bladder tumor cell proliferation and repressed CDDP-mediated apoptosis via regulation of PPP2R2A/AKT/mTOR axis. The AKT/mTOR pathway was markedly induced in bladder tumor cells with miR-222 upregulation. MiR-222 inhibited autophagy via activating the AKT/mTOR axis in bladder tumor cells [60]. GSK3 is one of the main substrates of AKT that inhibits GSK3 by phosphorylation [61]. Long non-coding RNAs (lncRNAs) interact with mRNAs, proteins, and miRNAs to regulate tumor progression [62]. They are also involved in regulation of chemoresistance and autophagy [63, 64]. The AC023115.3 upregulation has been observed in cisplatin-exposed glioma cells that stimulated the cisplatin-mediated apoptosis by autophagy inhibition. It also upregulated GSK3β and decreased autophagy through the miR-26a sponging [65].

HER3 is a receptor tyrosine kinase (RTK) that regulates cell growth and proliferation via the PI3K/AKT pathway [66]. It has been shown that there was significant miR-205 down regulation in nasopharyngeal cancer (NPC) cells. MiR-205 reduced the NPC cell proliferation while induced autophagy via LC3B II up regulation and p62 down regulation. MiR-205 increased CDDP sensitivity by HER3 targeting [67]. VEGFA is a growth factor that regulates cell proliferation via PI3K/AKT pathway. Circ_0023404 promoted the cervical cancer progression through miR-5047/VEGFA axis. It also induced CDDP resistance via inhibition of autophagy-mediated apoptosis in cervical tumor cells [50]. Transforming growth factor (TGF)-α is an EGF-like protein that functions as a ligand for the EGFR along with amphiregulin and EGF [68]. It was reported that miR-144 inhibited autophagy while enhanced CDDP sensitivity via TGF-α targeting in anaplastic thyroid cancer (ATC) cells [69]. CircEIF6 upregulated the TGF-α and increased cisplatin-resistance via autophagy enhancement through miR-144-3p sponging in thyroid tumor cells [70].

PRKCI belongs to the protein kinase C family that regulates tumor progression and chemosensitivity via regulation of the immune microenvironment and the WNT signaling pathway [71, 72]. CircPARD3 is an autophagy-suppressive circRNA abundantly expressed in LSCC tissues that was correlated with poor prognosis. It triggers proliferation, invasion, migration, and chemoresistance via suppression of autophagy in LSCC cells. CircPARD3 stimulated the AKT-mTOR axis and inhibited autophagy by upregulation of PRKCI via miR-145-5p sponging. There was a significant correlation between p62 upregulation and poor prognosis in LSCC patients. PRKCI was found to suppress autophagy while increased mTOR and AKT phosphorylation in LSCC cells [73]. Exosomes are currently being studied extensively due to their role in mediating miRNA transfer, which helps various malignancies resist chemotherapy [74, 75]. Additionally, exosomal miRNAs are regarded as intriguing biomarkers for determining treatment response or tracking disease advancement due to their accessibility from peripheral blood. The expression of miR-425-3p has been found to be triggered in exosomes and cells by c-Myc-mediated transactivation following cisplatin exposure. MiR-425-3p promoted drug resistance via enhancing autophagy through AKT1 targeting. Cisplatin treatment was associated with the upregulation of c-Myc together with β-catenin in NSCLC cells. C-Myc was observed to positively regulate miR-425-3p expression through direct binding to its promoter region [76].

Beclin-1, as the first recognized mammalian autophagic protein, is implicated in autophagy initiation and modulation of several tumor cell signaling pathways [77]. It is a component of the PI3K complex that is involved in regulation of vesicle-trafficking. Beclin-1 interacts with the Bcl-2 anti-apoptotic protein to induce cell death [78]. Its deregulation has been associated with the prognosis in many cancer types [79, 80]. Additionally, upregulation of Beclin-1 led to increased drug-resistance and autophagy upon CDDP treatment [81]. MiR-30a-5p was shown to downregulate Beclin-1 and hence promotes the efficacy of VP16/DDP chemotherapy in lung cancer [82]. PVT1 expression levels were found to be positively associated with tumor volume, TNM staging, lymph node involvement, poor survival, and cisplatin sensitivity in NSCLC. PVT1 promoted the autophagy-mediated cisplatin resistance via miR-216b/Beclin-1 axis [83]. MiR-30a inhibited the beclin1 mediated to induce CDDP mediated apoptosis in tumor cells [81].

Dynamin-Related Protein 1 (DRP1) is considered as an upstream regulator of mitochondrial fission, whose inhibition could be an efficient cancer treatment strategy [84, 85]. Mitochondrial fission 1 (FIS1) enhances CDDP sensitivity by functioning as a receptor to attract DRP1 into the mitochondria [86]. AKAP1 serves as a scaffold for the delivery of PKA to outer mitochondrial membrane in order to regulate the phosphorylation state of target proteins [87]. RAB12 belongs to the Ras family that can operate as a potential autophagy activator by blocking mTORC1 signaling or promoting autolysosome development [88, 89]. It has been reported that there was significant miR-148a-3p down regulation in CDDP-resistant GC cells. MiR-148a-3p induced CASP-3 and CDDP mediated cell death. AKAP1 inhibited the CDDP-induced mitochondrial fission by mediating the DRP1 phosphorylation and inactivation. MTORC1 prevented CDDP-induced death of GC cells via promoting early autophagosome production by RAB12 inhibiting. Additionally, miR-148a-3p concurrently targeted RAB12 and AKAP1 to make GC cells more susceptible to CDDP treatment [90].

Rho-associated protein kinase (ROCK) is a kinase that enhances the invasive capacity of tumor cells through interacting with the actin filaments to induce stress fibers and focal adhesions [91]. ROCK is a negative regulator of PI3K/AKT through PTEN phosphorylation and activation [92]. Its knockdown improves the CDDP effectiveness and prevents tumor progression and metastasis [93]. ROCK1 is a member of the ROCK family that functions as a downstream mediator of Rho A when activated by GTP binding [94]. MiR-136-5p suppressed cell migration while enhanced CDDP sensitivity by ROCK1 targeting in HPSCC and LSCC. MiR-136-5p upregulation coupled with cisplatin treatment was shown to downregulate P62 and repress the AKT/mTOR axis [95]. ROCK2 is a serine-threonine kinase that determines cell shape and migration by influencing the cytoskeleton [96]. It has been observed that there was circCUL2 down regulation in GC tissues, which was associated with the tumor differentiation, lymph node metastasis, and TNM stage. CircCUL2 suppressed GC cell proliferation and migration via regulating ROCK2 through miR-142-3p sponging. CircCUL2 also modulated the CDDP sensitivity via inducing autophagy through the miR-142-3p/ROCK2 axis [97].

### Role of miRNAs in autophagy-mediated CDDP response by regulation of apoptosis and drug efflux

Apoptosis is the primary mechanism of cell death that is inhibited in tumor cells to resist against drug mediated DNA damage [98, 99]. Although, apoptosis is directly linked to cell death, autophagy has a dual effect on tumor cells [100]. Apoptosis can be modulated through the Bcl-2 protein family that involves multiple members such as Bcl-2, Bax, CASP-9 [101]. Bcl-2, which is a critical regulator of apoptosis, has been found to be upregulated in a wide range of human malignancies [102]. On the other hand, it suppresses autophagy via Beclin1 targeting [103]. Beclin1 uses its BH3 domain to interact with different homologs of Bcl-2, resulting in autophagy suppression [104]. It has been shown that miR-15a-3p increased cisplatin sensitivity by suppressing Bcl-2 that resulted in autophagy induction in NSCLC cells [105]. MiR-143 was shown to regulate the Bcl-2, Bax, and CASP-9 apoptotic genes, which in turn enhanced the cisplatin-mediated death in cervical tumor cells. Interestingly, the combined miR-143 and cisplatin treatment resulted in autophagy induction, cell cycle arrest, c-Myc downregulation, and cell migration suppression by vimentin down regulation. Therefore, application of miR-143 in conjunction with cisplatin may provide a potential therapeutic approach for cervical cancer patients [106]. MiR-7-5p was found to be upregulated in cisplatin-resistant cervical tumor cells, boosting energy production via targeting Bcl-2 and decreasing energy consumption through PARP-1 targeting. MiR-7-5p induced autophagy through Bcl-2 down regulation in cisplatin-resistant cells [107]. There was significant miR-30a down regulation in CDDP-resistant oral squamous cell carcinoma (OSCC) cells. Exosomal delivery of miR-30a was associated with enhanced CDDP response in OSCC cells by inhibiting autophagy and improving apoptosis through Beclin1 and Bcl-2 targeting, respectively [108].

P-glycoprotein (P-gp) is a transmembrane ABC transporter that acts as an efflux pump to discharge a variety of chemotherapy drugs from MDR tumor cells [109]. It also protects chemo-resistant tumor cells toward the caspase-dependent apoptosis [110]. It has been shown that miR-30a inhibited the GC cell proliferation and CDDP resistance by P-gp and MDR1 down regulations. CDDP treatment promoted autophagy while reduced apoptosis in the SGC7901/CDDP-resistant cells. MiR-30a was correlated with CDDP resistance-related autophagy in SGC7901 cells [111]. MTDH is involved in autophagy and chemoresistance by MDR1 up regulation [112]. MTDH was suggested to promote 5-FU resistance by inducing autophagy via AMPK/ATG5 axis [113]. Osteosarcoma (OS) is primarily managed with different approaches, including surgical resection, radiotherapy, neoadjuvant chemotherapy, and adjuvant chemotherapy [114]. High-dose cisplatin, doxorubicin, etoposide, and methotrexate are frequently used in chemotherapeutic regimens [115]. MiR-22 has been observed to improve the efficiency of CDDP treatment which in turn suppressed the proliferation of osteosarcoma cells via downregulation of the autophagy-related genes, including beclin1, ATG5, and LC3. It also diminished CDDP resistance via blocking autophagy. Moreover, miR-22 suppressed MTDH upregulation induced by CDDP [116]. Another study also reported that miR-22 suppressed autophagy and cell proliferation while triggered CDDP sensitivity by targeting MTDH in OS cells [117].

### Role of miRNAs in autophagy-mediated CDDP response by regulation of ubiquitin-like modifiers and autophagy receptors

Autophagy is a cellular process that employs lysosomal machinery to recycle dysfunctional long half-life proteins and organelles. It begins with the creation of double membrane-bound vesicles known as autophagosomes and is regulated via conserved autophagy-related proteins [118]. It is vitally involved in tumor progression through autophagy-related (ATG) proteins [119]. Light chain 3 (LC3) is a critical autophagosome biomarker in the autophagy system which serves in substrate selection and autophagosome formation [120]. The cleavage of LC3 into the LC3-I variant with an exposed C-terminal glycine that permits association with phophatidylethanolamine to generate LC3-II, crucially involves in autophagosome formation. In addition, LC3 recycling occurs when LC3-II gets deconjugated from LC3-I through this proteolytic cleavage [121]. The p62 protein breaks down during autophagy and builds up as the autophagy declines [122]. The p62/LC3 interaction is one of the main ways to deliver the autophagic cargo [123]. It has been reported that miR-199a-5p has an important role in cisplatin resistance of SCLC via regulation of p62 mediated autophagy [124]. Cisplatin upregulates the UPR-related chaperones, including CALR, GRP78, and PDIA3, through inducing endoplasmic reticulum (ER) stress in tumor cells [125]. Conversely, ER stress tolerance (ERST) develops after repeated activation of the ER stress response by chemotherapeutic agents. CHOP is regarded as a crucial regulator of apoptosis induced by ER stress [126, 127]. Nutrient deficiency, UV rays, tunicamycin, and thapsigargin promote ER stress and CHOP expression [128–130]. CHOP was shown to be significantly downregulated in patients with recurrent lung cancer compared to those without recurrence that was associated with a worse overall survival rate. CHOP expression was also accompanied by an increase in the levels of DR5, LC3-II, and TRB3. Moreover, miR-146a induced chemoresistance by CHOP targeting in lung tumor cells [131]. YES1 has been characterized as an oncogene that could serve as a therapeutic target in several malignancies [132]. It was demonstrated that ovarian cancer patients with Yes1 up regulation had more sensitivity to platinum and a better prognosis compared with down regulated patients [133]. MiR-133a decreased CDDP resistance via targeting YES1 and autophagy regulation in ovarian tumor cells. YES1 induced CDDP resistance by upregulating LC3B in a xenograft tumor model [134].

### Role of miRNAs in autophagy-mediated CDDP response by regulation of transcription factors and DNA binding proteins

Forkhead box gene P1 (FOXP1) is a transcription factor involved in embryogenesis and myocardial development [135]. FOXP1 was considered to be implicated in CDDP resistance by targeting ATG14 in ovarian cancer. MiR-29c-3p impeded autophagy and CDDP resistance via FOXP1/ATG14 axis in ovarian tumor cells [136]. It has been observed that miR-125b promoted autophagy in FTC and ATC cells by Foxp3 targeting. MiR-125b induced drug sensitivity to sorafenib and cisplatin in thyroid cancer cells. Autophagy was also induced by upregulation of ATG7 and LC3II and downregulation of Bcl-2 via Foxp3 suppression. Although, miR-125b or cisplatin could individually shrink the tumor size, the combination of miR-125b and cisplatin showed the most pronounced antitumor activity in a xenograft mouse model [137]. Early growth response factor 1 (EGR1) is a transcription factor that plays a pivotal role in transcriptional induction of the apoptotic pathway regulators such as TP53, TNF, TP53, BAX, and RB1 following chemotherapy or radiotherapy [138]. EGR1-MIR152 was shown to regulate cisplatin-mediated autophagy in ovarian tumor cells via ATG14 targeting. MiR-152 suppressed cisplatin-induced autophagy in tumor cells. EGR1 regulated miR-152 and ATG14, which then improved CDDP sensitivity in ovarian tumor cells [139].

CCCTC-binding factor (CTCF) is a zinc finger transcription factor that participates in several gene regulatory mechanisms by the chromatin structure modulation [140, 141]. CTCF as a key transcription factor influences tumor progression via regulation of lncRNAs [142, 143]. MSH6 is a MutS family member that is involved in the eukaryotic mismatch repair system by heterodimerizing with MSH2 to build a mismatch recognition complex [144]. CTCF upregulated IGF2-AS to promote autophagy by upregulating Beclin1, resulting in greater CDDP resistance in osteosarcoma cells. IGF2-AS increased CDDP resistance in OS cells by upregulating MSH6 via miR-579-3p sponging [145].

High mobility group box-1 (HMGB1) is a nucleoprotein that has key roles in regulation of DNA replication, damage repair, and apoptosis [146]. HMGB1 upregulation has been reported in numerous malignancies that were associated with poor prognosis and metastasis [147]. In addition, HMGB1 as an essential regulator of autophagy is also involved in chemo resistance of tumor cells [148]. A majority of tumor cells release HMGB1 during externally induced apoptosis and autophagy. Moreover, HMGB1 silencing was shown to sensitize tumor cells to chemo-radio therapeutic modalities [149]. HMGB1 also activate autophagy in response to stress by detaching Beclin-1 from Bcl-2 [150]. H19 knockdown significantly inhibited autophagy and limited CDDP resistance through regulation of miR-107/HMGB1 axis in LSCC cells [151]. Vitamin D receptor (VDR) is a nuclear receptor that regulates autophagy and a number of genes implicated in cell proliferation, differentiation, and calcium-phosphate homeostasis [152, 153]. MiR-181a-5p enhanced autophagy and CDDP sensitivity in breast tumor cells via VDR targeting [154].

### Role of miRNAs in autophagy-mediated CDDP response by regulation of autophagy-related genes

**Fig. 2:**
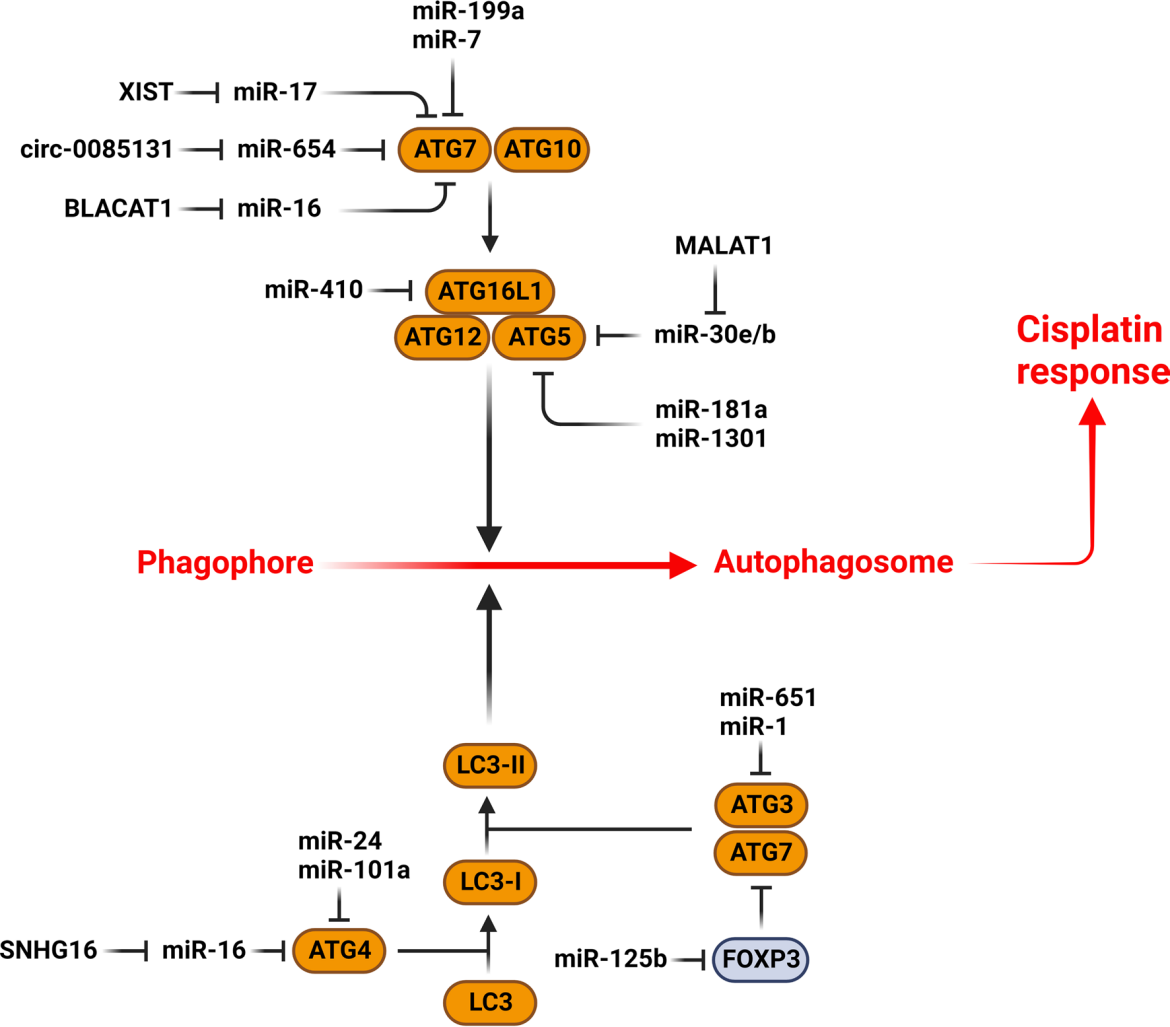
Role of miRNAs in autophagy-mediated cisplatin response via regulation of autophagy-related genes (ATGs). (Created with BioRender.com)

Autophagy is involved in elimination of the endogenous substances through lysosomal degradation pathway, which is regulated by autophagy-related genes (ATGs) [155]. MicroRNAs have a pivotal role in autophagy-mediated cisplatin reponse by regulation of ATG proteins (Fig. 2). ATG2B is a member of the ATG2 family, which interacts with WIPI4 and GABARAP to generate phagophores and transport lipids [156–158]. ATG2B participates in the early stages of autophagosome assembly via associating with WDR45 and ATG2A [159]. It stimulates differentiation of the hematopoietic progenitor cells by making them more sensitive to thrombopoietin [160]. There was significant miR-1278 down regulation in NPC tissues that was correlated with poor chemotherapy outcomes and overall survival. MiR-1278 down regulated ATG2B to suppress autophagy and increase CDDP sensitivity in NPC cells [161]. It has been reported that there was significant miR-375 down regulation in CDDP resistant OS cells. It enhanced cell death while inhibited cell proliferation and autophagy through ATG2B targeting in CDDP-resistant OS cells [162]. MiR-376a was found to be involved in drug resistance conferred by circPGAM1 in laryngeal carcinoma. CircPGAM1 increased autophagy-mediated CDDP resistance by regulation of the miR-376a/ATG2A axis [163]. There was miR-1 down regulation in CDDP resistant NSCLC tissues. MiR-1 was inversely correlated with LC3B autophagy factor in NSCLC tissue samples. MiR-1 blocked autophagy-mediated by ATG3 and thus enhanced CDDP sensitivity in NSCLC cells [164]. It was shown that miR-651 reduced the CDDP resistance of cervical tumor cells by ATG3 targeting [165].

ATG4 is a cysteine protease that functions as an oncogene by promotion of autophagy. Meanwhile, its phosphorylation could inhibit tumor cell function and decrease autophagy [166, 167]. It has been shown that SNHG16 promoted the osteosarcoma cell survival, invasion, and autophagy-mediated chemo resistance via miR-16/ATG4B axis [168]. MiR-101-3p was reported to inhibit CDDP-mediated autophagy via ATG4D targeting in NSCLC cells. MiR-101a-3p and cisplatin therapy upregulated p62 while suppressed the LC3II/LC3I ratio and the formation of autolysosomes and autolysosomes in NSCLC cells [169]. Autophagy protects liver tumor cells from cell death mediated by anti tumor drugs. MiR-101 increased CDDP-mediated apoptosis and suppressed autophagy by ATG4D and mTOR targeting in HCC cells [170]. It has been reported that miR-24-3p inhibited autophagy and promoted the VP16-DDP response via targeting ATG4A in SCLC [171].

ATG5 is involved in autophagy via ATG12 and LC3 (ATG8) conjugation to produce autophagosomes [172]. Chemo-resistant GC cells had elevated levels of autophagy compared with chemo-sensitive GC cells. Propofol increased CDDP-mediated apoptosis via regulation of MALAT1/miR-30e/ATG5 axis in GC cells [173]. MiR-181a suppressed autophagy through ATG5 targeting in CDDP resistant GC cells [174]. It was observed that there was significant miR-1301 up regulation in ovarian tumor-resistant cells. MiR-1301 induced cell invasion and proliferation and up regulated the EMT-related genes such as Slug, Snail, and N-cadherin while down regulated E-cadherin, Beclin1, and ATG5 [175]. It has been observed that MALAT1 enhanced CDDP resistance via autophagy activation through regulation of the miR-30b/ATG5 pathway in CDDP-resistant GC cells [176].

Autophagy-related protein 7 (ATG7) is a critical positive regulator of autophagy by stimulating the ATG8 and ATG12 [177, 178]. The cell cycle alteration brought on by ATG7-mediated autophagy has been shown to promote the generation of neural crest cells [179]. ATG7 has been found to be up-regulated in various malignancies, correlating with tumor chemoresistance [180, 181]. TRIM65 E3 ubiquitin ligase is involved in regulation of autophagy, immunity, carcinogenesis, and chemoresistance [182–184]. TRIM65 down regulation suppressed autophagy while induced CDDP-mediated apoptosis via LC3-II and ATG7 down regulations in lung tumor cells. MiR-138-5p regulated the role of TRIM65 in CDDP resistance and autophagy [185]. A significant down regulation of miR-199a-5p has been shown in HCC patients undergoing cisplatin treatment, which improved autophagy via ATG7 targeting. Downregulated miR-199a-5p in cisplatin-treated HCC cells enhanced CDDP resistance through autophagy induction [186]. Tissue samples taken from cisplatin-resistant NSCLC patients showed circ_0085131 up regulation that was correlated with poor prognosis. There was a positive association between the expression levels of ATG7 and circ_0085131 in NSCLC cells. ATG7 played a pivotal oncogenic role in cisplatin-resistance of NSCLC patients. Circ_0085131 induced cisplatin resistance via miR-654-5p/ATG7 axis in NSCLC cells [187]. BLACAT1 up regulation has been observed in CDDP-resistant NSCLC cells. It increased the resistance of NSCLC cells to CDDP by enhancing autophagy through the miR-17/ATG7 axis [188]. MiR-7-5p inhibited the autophagy through ATG7 targeting in BCa. ATG7 was shown to diminish miR-7-5p-induced CDDP sensitivity in BCa cells [189]. It has been reported that there was lncRNA-XIST up regulation in NSCLC tissues and CDDP-resistance A549 cells that was correlated with TNM stage. LncRNA-XIST induced cisplatin resistance by activating autophagy through the miR-17/ATG7 axis in NSCLC cells [180]. Circ-PKD2 promoted autophagy and CDDP sensitivity by targeting miR-646 in OSCC cells. It also enhanced apoptosis by miR-646/ATG13 axis that up regulated CASP-8 in OSCC cells [190]. SNHG14 promoted CRC cisplatin resistance by inducing autophagy through regulation of the miR-186/ATG14 axis [191]. Autophagy impedes the effectiveness of cisplatin, doxorubicin, and methotrexate in osteosarcoma cells [192–194]. ATG16L1 is a member of a multimeric complex required for autophagy. It has been shown that miR-410 improved CDDP sensitivity by suppressing autophagy via ATG16L1 targeting in osteosarcoma cells [195].

### Role of miRNAs in autophagy-mediated CDDP response by regulation of structural factors and enzymes

LAMP3 is a lysosomal membrane glycoprotein that has a key role in protein degradation and lysosome-autophagosome fusion [196, 197]. LAMP3 was found to be activated subsequent to the induction of the PERK/eIF2a/ATF4 axis in unfolded protein response (UPR) pathway [198]. MiR-205 was indicated to impede the autophagy by promoting the lysosomal disruption through down-regulating LAMP3 that enhaced cisplatin sensitivity due to interfering with the detoxification function of PCa cells [199]. WEE1 is a Ser/Thr kinase that inhibits the CDC2/cyclin B during cell cycle progression. FGD5-AS1 or WEE1 inhibition reduced CDDP-resistant cell viability and autophagy while promoted apoptosis in NSCLC. FGD5-AS1 induced NSCLC cell progression and CDDP resistance via miR-140-5p/WEE1 axis [200]. The motor protein myosin-X (MYO10) is significantly up regulated in metastatic tumors [201]. It connects integrins to microtubules in order to induce filopodia construction [202]. SNHG7 was found to increase cisplatin-mediated autophagy via regulating the miR-329-3p/MYO10 axis in NB cells [203]. SIRT1 belongs to the sirtuin family of histone deacetylases that is involved in a wide range of cellular mechanisms, including cell cycle, survival, metabolism, aging, chemoresistance, and apoptosis [204, 205]. SIRT1 has been observed to exhibit tumor-suppressive or oncogenic roles in various malignancies [206, 207]. It has been reported that miR-124 and miR-142 inhibited autophagy while induced apoptosis via SIRT1 targeting in CDDP-resistant NSCLC cells [208].

## Conclusions

Cisplatin is considered as one of the most common first-line chemotherapy drugs in metastatic tumors. However, there is a noticeable rate of CDDP resistance in cancer patients. Autophagy is considered as one of the main causes of CDDP resistance in tumor cells. Therefore, autophagy regulatory factors can be involved in CDDP response. Considering the role of miRNAs in the regulation of autophagy, in the present review we discussed the role of miRNAs in CDDP response. It has been reported that miRNAs mainly increase the CDDP sensitivity through inhibition of autophagy. Therefore, this review can be an effective step to suggest miRNAs as the efficient therapeutic options to enhance the autophagy-mediated CDDP sensitivity in tumor cells.

## Data Availability

The datasets used and/or analyzed during the current study are available from the corresponding author on reasonable request.

## Abbreviations

ATG5: Autophagy related 5
ATC: Anaplastic thyroid cancer
ATGs: Autophagy-related genes
ATG7: Autophagy-related protein 7
CTCF: CCCTC-binding factor
circRNAs: Circular RNAs
CDDP: Cisplatin
DRP1: Dynamin-related protein 1
EGR1: Early growth response factor 1
ER: Endoplasmic reticulum
ERST: ER stress tolerance
FOXP1: Forkhead box gene P1
GC: Gastric cancer
HMGB1: High mobility group box-1
LC3: Light chain 3
lncRNAs: Long non-coding RNAs
miRNAs: MicroRNAs
LC3: Microtubule-associated protein light chain
MYO10: Motor protein myosin-
MDR: Multi-drug resistance
NPC: Nasopharyngeal cancer
NSCLC: Non-small-cell lung cancer
OSCC: Oral squamous cell carcinoma
OS: Osteosarcoma
P-gp: P-glycoprotein
PP2A: Phosphatase 2A
PI3P: Phosphatidylinositol 3-phosphate
RTK: Receptor tyrosine kinase
ROCK: Rho-associated protein kinase
TGF: Transforming growth factor
UPR: Unfolded protein response
VDR: Vitamin D receptor
